# Multi-User Scheduling for 6G V2X Ultra-Massive MIMO System

**DOI:** 10.3390/s21206742

**Published:** 2021-10-11

**Authors:** Shibiao He, Jieru Du, Yong Liao

**Affiliations:** 1 School of Electronic Information, Chongqing Institute of Engineering, Chongqing 400056, China; 04116@cqie.edu.cn; 2 School of Microelectronics and Communication Engineering, Chongqing University, Chongqing 400044, China; 202012021021t@cqu.edu.cn

**Keywords:** 6G V2X, precoding, matrix correlation, noise enhancement factor, multi-user scheduling

## Abstract

6G vehicle-to-everything (V2X) communication will be combined with vehicle automatic driving technology and play an important role in automatic driving. However, in 6G V2X systems, vehicle users have the characteristics of high-speed movement. Therefore, how to provide stable and reliable wireless link quality and improve channel gain has become a problem that must be solved. To solve this problem, a new multi-user scheduling algorithm based on block diagonalization (BD) precoding for 6G ultra-massive multiple-input multiple-output (MIMO) systems is proposed in this paper. The algorithm takes advantage of the sensitive nature of BD precoding to channel correlation, uses the Pearson coefficient after matrix vectorization to measure the channel correlation between users, defines the scheduling factor to measure the channel quality according to the user noise enhancement factor, and jointly considers the influence of the correlation between user channels and channel quality, ensuring the selection of high-quality channels while minimizing channel correlation. Simulation results show that compared with the multi-user scheduling algorithm based on subspace correlation, condition number, and geometric angle, the proposed algorithm can obtain higher user channel gain, effectively reduce the system bit error rate, and can be applied to 6G V2X communication.

## 1. Introduction

Vehicles to Everything (V2X) is a large interactive network composed of vehicle information such as vehicle location and speed, which includes four types of communication: vehicle-to-vehicle (V2V), vehicle-to-infrastructure (V2I), vehicle-to-network (V2N), and vehicle-to-pedestrian (V2P). V2X establishes a platform for intelligent information interaction between people, vehicles, and roads, and finally realizes the deep integration of people, vehicles, and the environment [1]. At present, automatic driving vehicle terminals are equipped with many sensors, which can assist the vehicle to obtain environmental information, expand the identification range of existing sensors by using the external information received by V2X communication, support safer and natural automatic driving, and realize intelligent driving function. With the development of 6G networks, 6G V2X is an effective technology that can meet the needs of automatic driving and can support some conditions for automatic driving [2,3]. Ultra-massive MIMO technology is proposed to support higher capacity communication in 6G networks. It is similar to the concept of 5G massive MIMO and further increases the scale of transmitting antennas. In theory, more antennas lead to exponential growth of system capacity, which can further improve energy efficiency and greatly increase the throughput of wireless communication systems. Ultra-massive MIMO technology can well meet the needs of 6G high transmission rates and highly reliable communication [4,5,6,7]. However, ultra-massive MIMO technology needs to face new problems brought by the sharp increase in the number of antennas, densification, and beam directionality, such as limited system resources and reliable communication of dynamic users [8]. Therefore, it is of great significance for 6G V2X communication to design an algorithm that can solve the problem of limited system resources in an ultra-massive MIMO system.

In recent years, much research work has been devoted to improving the transmission capability of communication systems. A scheme in which the antenna selection of the base station and the user scheduling at the receiver side are performed simultaneously was proposed in [9]. This scheme employs an algorithm based on semi-orthogonal user selection for user scheduling. The main goal of the combination of antenna selection and user scheduling algorithms is to maximize the sum-rate capacity of the system; the selected group of antennas is used to transmit data to the best user set selected by the user scheduling algorithm. In the 6G V2X scenario, the channel is time-varying, so the feasibility of this method is low. A novel cyclic user grouping technology to reduce the complexity of the system was proposed in [10], using three different combinations of user selection schemes and semi-orthogonal user selection based on the norm-based antenna scheduling method to achieve higher system capacity. The existing techniques in the development of massive MIMO specifically concentrating on energy and spectral efficiency was analyzed and the challenges in the system design were discussed in [11]. A detailed investigation of the existing energy efficiency of massive MIMO system design and its preliminary and state-of-the-art technologies was conducted to explore energy-saving MIMO systems for 5G communications. It was pointed out that in multi-user and multi-cell environments, the existence of inter-user and inter-cell interference complicates the design of energy-saving MIMO systems. The idea of using space resources to maximize energy efficiency while suppressing interference is worth studying. Antenna selection and user scheduling were studied in a down-link multi-user MIMO system, and the aims were to maximize users sum rate and reduce complexity in [12]. In this investigation, a low computational complexity sub-optimal user scheduling scheme based on matrix Gauss elimination method was combined with maximum norm antenna selection to maximize users sum rate and reduce complexity, under the constraints based on the number of maximum service users and the maximum transmission power. The proposed algorithm gained much improved sum rate performance over the semi-orthogonal user scheduling algorithm and had lower complexity compared to the exhaustive search algorithm. Aiming at the maximum minimum fairness precoding problem under the antenna power constraint, a suboptimal precoding algorithm was proposed by decoupling the problem into a precoding direction vector design problem and a power allocation problem in [13]. The signal-to-leakage-and-noise ratio (SLNR) metric was considered in the precoding direction vector design to properly mitigate the inter-group interference. Moreover, aiming at the maximum minimum fairness criterion, the power allocation procedure can guarantee fairness between users. However, the above-noted designs of the user scheduling algorithms were based on static user channels, and did not consider the real situation of channel dynamic changes.

In view of the above problems, we propose a 6G-oriented new multi-user scheduling algorithm based on block diagonalization (BD) precoding for ultra-massive MIMO systems. The main work of this paper is as follows:

(1) Based on the high-speed movement of 6G V2X and the characteristics of millimeter waves, the time-varying geometric channel model is adopted. Doppler frequency shift is generated due to the high-speed moving characteristics of the vehicle. The channel is more suitable for the research scenario in this paper, because the Doppler frequency shift is introduced into the channel model and combined with the conventional millimeter wave channel.
(2) On the basis of the time-varying channel model, for the BD precoding algorithm’s sensitivity to channel correlation, the Pearson coefficient between users and the user noise enhancement factor are jointly considered, and the Pearson coefficient after matrix vectorization is used to measure the channel correlation between users The scheduling factor that can measure the quality of the channel is designed according to the user noise enhancement factor. It satisfies the minimum user interference while ensuring the channel quality of the scheduled users. This algorithm overcomes the operational problems of using many matrix decompositions in the scheduling algorithm, effectively reduces the system error rate, and improves the system throughput rate.

The purpose of this paper is to propose a multi-user scheduling algorithm for the ultra-massive MIMO system in the 6G V2X scenario. Section 2 introduces the 6G V2X ultra-massive MIMO system model based on multi-user scheduling and the channel model suitable for car networking scenarios. Section 3 introduces BD precoding and provides a multi-user scheduling algorithm based on BD precoding. The proposed algorithm is simulated and analyzed in Section 4. The paper concludes with some final remarks in Section 5.

## 2. System Model

### 2.1. G V2X Ultra-Massive MIMO System Model Based on Multi-User Scheduling

**Figure 1:**
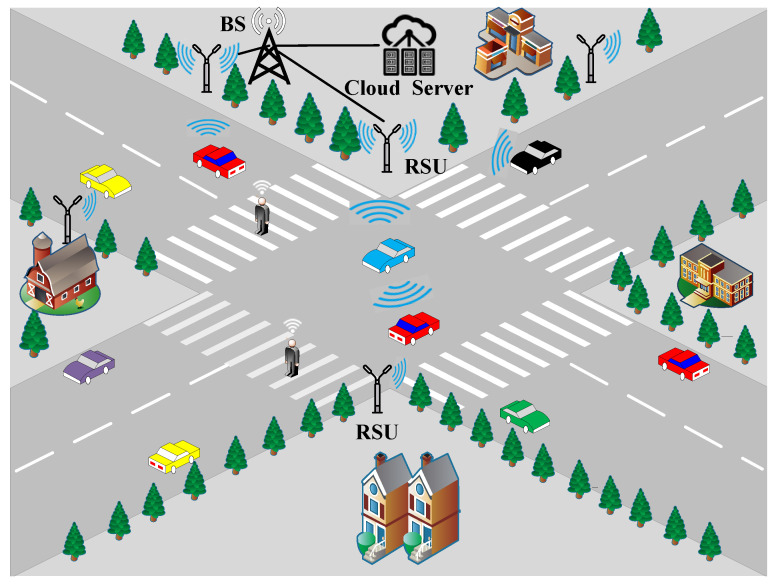
6G V2X classic communication scenario.

**Figure 2:**
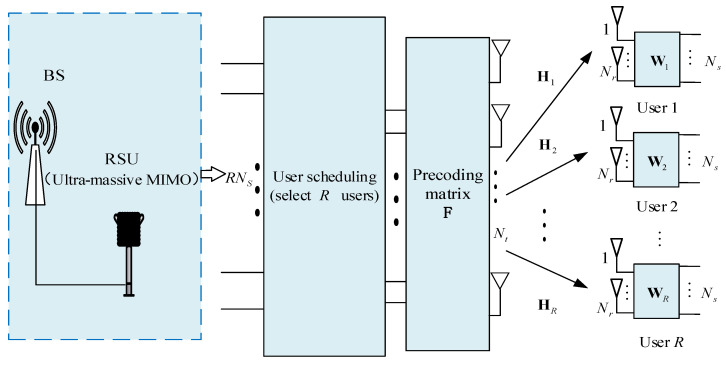
Multi-user scheduling for ultra-massive MIMO systems.

In this section, we consider a typical 6G V2X communication scenario, as shown in Figure 1, and the ultra-massive MIMO downlink system in the 6G V2X autonomous scenario shown in Figure 2. The system consists of a base station (BS), a roadside unit (RSU), and vehicles connected to the base station (BS). BS and RSU are connected by optical fiber. The RSU (transmitting end) is equipped with $N_{t}$ antennas and the set of vehicles is N={1, 2, …, k}, where each vehicle user is equipped with $N_{r}$ receiving antennas and multiple sensors. The system can schedule R={1, 2, …, R},R⊂N vehicles each time. In the downlink of the system with the assumption that the channel state information (CSI) is available, the maximum number of transmission data streams that each user can support is $N_{s}$, the total number of data streams sent by the base station is $RN_{s}$.

This paper assumes that the user channel information is known at the transmitter and receiver. In the time division duplex mode, the base station can obtain the user’s channel information through uplink channel estimation. In the frequency division duplex mode, at the beginning of each communication time slot, the user uses the feedback link to obtain the user’s channel information. The channel information is fed back to the transmitting end, and the transmitting end selects and designs the corresponding precoding matrix for users based on the channel feedback information and then communicates with the selected users.

It can be seen from Figure 2 that the original signal is transmitted through the precoder at the antenna end and received by the receiving antenna of the receiving end through the channel. The received signal passes through the combiner to eliminate the interference between the user sub-channels, and finally undergoes signal processing to obtain the transmitted signal vector.

At the BS side, the transmitted data information is given as

(1) $$s=\sum_{k=1}^{R}F_{k}s_{k}$$

where $s\in {\mathbb{C}}^{(R\times N_{s})\times 1}$ denotes the original transmission symbol vector and is such that $E\{ss_{}^{H}\}=I_{RN_{S}}$, $s_{k}\in {\mathbb{C}}^{N_{s}\times 1}$ is the original signal vector sent by the RSU to user k with $E\{s_{k}s_{k}^{H}\}=I_{N_{s}}$, and $F_{k}\in {\mathbb{C}}^{N_{t}\times N_{s}}$ is the precoding matrix of user k with ${\left\|F_{k}\right\|}^{2}=1$, which is the unitary matrix.

If the vehicle has perfect time and frequency synchronization for each time slot t, the received signal of user k after passing through the channel is expressed as

(2) $$y_{k}\left(t\right)=H_{k}\left(t\right)F_{k}s_{k}+\sum_{i=1,i\neq k}^{R}H_{k}\left(t\right)F_{i}s_{i}+n_{k}\left(t\right),k=1,2,\ldots R$$

where the first item denotes useful data for user k, $H_{k}\left(t\right)\in {\mathbb{C}}^{N_{r}\times N_{t}}$ is the channel matrix between the RSU and user k in the $t^{th}$ time slot, and each element of the matrix obeys a Gaussian distribution with zero mean and unit variance. The second item denotes interference from other user data to the user data. The third item $n_{k}\left(t\right)\in {\mathbb{C}}^{N_{r}\times 1}$ is additive white Gaussian noise of $CN\left(0,\sigma_{k}^{2}I\right)$.

The received signal obtained after the combiner is given as

(3) $$\overset{}{{\tilde{y}}_{k}\left(t\right)}=W_{k}{}^{{}^{H}}H_{k}\left(t\right)F_{k}s_{k}+\sum_{i=1,i\neq k}^{R}W_{k}{}^{{}^{H}}H_{k}\left(t\right)F_{i}s_{i}+W_{k}{}^{H}n_{k}\left(t\right),k=1,\;2,\;\ldots R$$

where $W_{k}$ is combiner of user k.

If proper precoding is used, the data interference between users can be eliminated at the receiving end, and Formula (3) is expressed as

(4) $$\overset{}{{\tilde{y}}_{k}\left(t\right)}=W_{k}{}^{{}^{H}}H_{k}\left(t\right)F_{k}s_{k}+W_{k}{}^{H}n_{k}\left(t\right),k=1,\;2,\;\ldots R$$

### 2.2. Channel Model

Due to the high-speed movement of the vehicle, the channel characteristics are time-varying, resulting in a Doppler effect. Therefore, introducing the Doppler effect into the channel model and combining it with the conventional millimeter wave channel, the channel of the $t^{th}$ slot is expressed as a time-varying geometric channel model [14].

(5) $$H\left(t\right)=\gamma \sum_{i=1}^{N_{c}}\sum_{l=1}^{N_{ray}}\alpha_{il}\left(t\right)a_{r}\left(\theta_{il}\left(t\right)\right)a_{t}^{H}\left(\varphi_{il}\left(t\right)\right)e^{j2\pi f_{il}T_{s}t}$$

where $\gamma =\sqrt{N_{t}N_{r}/N_{c}N_{ray}}$ denotes the normalized correction factor, $N_{c}$ is the number of clusters in the channel, and $N_{ray}$ is the number of sub-channels in each cluster. The expression $\alpha_{il}\left(t\right)∼CN\left(0,\;1\right)$ represents the gain of the $i^{th}$ path in the *l*^th^ cluster. The expressions $a_{r}\left(\theta_{il}\left(t\right)\right)\in {\mathbb{C}}^{N_{r}\times 1}$ and $a_{t}\left(\varphi_{il}\left(t\right)\right)\in {\mathbb{C}}^{N_{t}\times 1}$ are the array steering vectors of the receiving and transmitting antennas, respectively.

(6) $$a_{r}\left(\theta_{il}\left(t\right)\right)={\left[1,e^{j\frac{2\pi }{\lambda }d\sin\left(\theta_{il}\left(t\right)\right)},\ldots ,e^{j\left(N_{r}-1\right)\frac{2\pi }{\lambda }d\sin\left(\theta_{il}\left(t\right)\right)}\right]}^{T}$$

(7) $$a_{r}\left(\varphi_{il}\left(t\right)\right)={\left[1,e^{j\frac{2\pi }{\lambda }d\sin\left(\varphi_{il}\left(t\right)\right)},\ldots ,e^{j\left(N_{r}-1\right)\frac{2\pi }{\lambda }d\sin\left(\varphi_{il}\left(t\right)\right)}\right]}^{T}$$

where the variables $\theta_{il}\left(t\right)\in [-\frac{\pi }{2},\frac{\pi }{2}]$ and $\varphi_{il}\left(t\right)\in [-\frac{\pi }{2},\frac{\pi }{2}]$ are the arrival angle and departure angle of the $i^{th}$ path in the *l*^th^ cluster, λ is the millimeter wave wavelength, and d is the distance between the antenna elements.

Assuming that the power is equally distributed, the system uses the BD precoding algorithm at the transmitting end. To get the channel capacity of user k,

(8) $$C_{k}=B\log_{2}\det\left(I_{N}+\frac{P}{M\sigma^{2}}H\left(t\right)H^{H}\left(t\right)\right)$$

The user set channel capacity can be expressed as

(9) $$\begin{array}{ll} C\left({\left[H_{i}^{H}\left(t\right)H_{j}^{H}\left(t\right)\right]}^{H}\right) & =B\log_{2}\det(I_{kN}+\\ & \frac{P}{K\sigma^{2}}{\left[H_{i}^{H}\left(t\right)H_{j}^{H}\left(t\right)\right]}^{H}\left[H_{i}^{H}\left(t\right)H_{j}^{H}\left(t\right)\right])\\ & =C_{i}+B\log_{2}\det(I_{N}+\frac{P}{K\sigma^{2}}H_{j}\left(t\right)H_{j}^{H}\left(t\right)-\\ & {\left(\frac{P}{K\sigma^{2}}\right)}^{2}H_{j}\left(t\right)H_{i}^{H}\left(t\right)\;{\left(I_{N}+\frac{P}{K\sigma^{2}}H_{j}\left(t\right)H_{j}^{H}\left(t\right)\right)}^{-1}H_{i}\left(t\right)H_{j}^{H}\left(t\right))\; \end{array}$$

## 3. Ultra-Massive MIMO Multi-User Scheduling Based on BD Precoding

The BD algorithm has been widely studied in ultra-massive MIMO systems, but it does not consider the channel correlation and channel conditions. The user scheduling algorithm with the optimal channel correlation is the exhaustive method, which traverses the user set to be selected and selects the user with the lowest correlation. The channel correlation cannot be used as the only criterion for selecting the optimal user set. It also needs to consider the quality of the user’s own channel conditions, because the user’s own channel conditions are also a key factor for the system. Therefore, an ultra-massive MIMO multi-user scheduling algorithm based on channel correlation and channel conditions under BD precoding scheme is proposed.

### 3.1. BD-Based Precoding

BD precoding is a linear precoding widely recognized in the downlink of ultra-massive MIMO systems; the core of its algorithm is to perform two Singular Value Decomposition (SVD) to obtain the precoding matrix F in which the user’s precoding matrix of user k is $F_{k}=F_{{}_{k}}^{1}F_{{}_{k}}^{2}$.

Define the system channel matrix as

(10) $$H={\left[H_{1}^{H},H_{2}^{H},\ldots ,H_{R}^{H}\right]}^{H}$$

The complement matrix ${\tilde{H}}_{k}$ of user k is given as

(11) $${\tilde{H}}_{k}={\left[H_{1}^{H},\ldots ,H_{k-1}^{H},H_{k+1}^{H}\ldots ,H_{R}^{H}\right]}^{H}$$

Using SVD decomposition of ${\tilde{H}}_{k}$, we can get

(12) $${\tilde{H}}_{k}={\tilde{U}}_{K}\left[\begin{array}{l} T_{k}\;0\\ 0\;0 \end{array}\right]{\left[{\tilde{V}}_{k}^{\left(1\right)}\;{\tilde{V}}_{k}^{\left(0\right)}\right]}^{H}$$

where ${\tilde{V}}_{k}^{\left(1\right)}\in {\mathbb{C}}^{N_{t}\times {\tilde{L}}_{k}}$ and ${\tilde{V}}_{k}^{\left(0\right)}\in {\mathbb{C}}^{N_{t}\times (N_{t}-{\tilde{L}}_{k})}$ are the first ${\tilde{L}}_{k}=rank\left({\tilde{H}}_{k}\right)$ and the last $\left(N_{t}-{\tilde{L}}_{k}\right)$ right singular value vectors of ${\tilde{H}}_{k}$, and ${\tilde{V}}_{k}^{\left(0\right)}$ is in the zero space of the complement matrix ${\tilde{H}}_{k}$.

Let $F_{k}^{1}={\tilde{V}}_{k}^{\left(0\right)}$, the system equivalent channel matrix ${\bar{H}}_{eqk}\in {\mathbb{C}}^{N_{r}\times (N_{t}-{\tilde{L}}_{k})}$ is obtained based on SVD decomposition

(13) $${\bar{H}}_{eqk}=H_{k}{\tilde{V}}_{k}^{\left(0\right)}=U_{k}\left[\begin{array}{cc} {\tilde{T}}_{k} & 0\\ 0 & 0 \end{array}\right]\left[\begin{array}{cc} {\bar{V}}_{k}^{\left(1\right)} & {\bar{V}}_{k}^{0} \end{array}\right]$$

Then, let $F_{k}^{2}={\bar{V}}_{k}^{\left(1\right)}\in {\mathbb{C}}^{(N_{t}-R_{1})\times N_{s}}$ and $U_{k}\in {\mathbb{C}}^{N_{r}\times N_{r}}$; we can get the precoding matrix and combiner of user k as

(14) $$F_{k}={\tilde{V}}_{k}^{\left(0\right)}{\bar{V}}_{k}^{\left(1\right)}$$

(15) $$W_{k}=U_{k}$$

Therefore, the main objective in the BD precoding algorithm is to find the optimal precoding matrix of user k to satisfy $H_{i}F_{k}=0(\forall i\neq k)$, to achieve the goal of eliminating interference.

### 3.2. Multi-User Scheduling Based on BD Precoding

Precoding is used to solve the problem of interference between users. However, after analyzing the BD precoding algorithm, we know that if there is a strong correlation between channels, a large part of their useful signals will be eliminated when eliminating the interference of other users. The received signal at the receiving end is weak and cannot be demodulated normally, which will greatly affect the capacity of the system. If the correlation between the user channel matrices is relatively low, this situation will not occur. The pre-coded users do not need to worry about interference from other users and do not have to worry about their own cancellation effects, and the system capacity can also be significantly improved. Therefore, the correlation between users can be used as part of the scheduling criteria to schedule each new user.

The theory proves that when the column vectors of the splicing matrix $H_{i,j}=[H_{i},H_{j}]$ composed of two matrices matrix $H_{i}$ and matrix $H_{j}$ are more orthogonal, the correlation between matrix $H_{i}$ and matrix $H_{j}$ is lower. Therefore, the Pearson coefficient after matrix vectorization is used in this paper to express the correlation of the user channel matrix. First, vectorize matrix $H_{i}$ and matrix $H_{j}$ respectively

(16) $$H_{i}\in C^{m\times n}\to HV_{i}\in C^{1\times (m\times n)}$$

(17) $$H_{j}\in C^{m\times n}\to HV_{j}\in C^{1\times (m\times n)}$$

The Pearson coefficient between vectors $HV_{i}$ and vectors $HV_{j}$ is given as

(18) $$\begin{array}{ll} coor_{ij}(HV_{i},HV_{j}) & =\frac{N\sum HV_{i}HV_{j}-\sum HV_{i}\sum HV_{j}}{\sqrt{N\sum HV_{{}_{i}}^{2}-(\sum HV_{i}{)}^{2}}\sqrt{N\sum HV_{{}_{j}}^{2}-(\sum HV_{j}{)}^{2}}}\\ & =\frac{\sum \left(HV_{i}-\bar{HV_{i}}\right)(HV_{j}-\bar{HV_{j}})}{\sqrt{{\sum \left(HV_{i}-\bar{HV_{i}}\right)}^{2}{\sum \left(HV_{j}-\bar{HV_{j}}\right)}^{2}}} \end{array}$$

where the absolute value of $coor(HV_{i},HV_{j})\in [-1,1]$ denotes the Pearson correlation coefficient of channel vector $HV_{i}$ and vector $HV_{j}$. When the absolute value of $coor_{ij}(HV_{i},HV_{j})$ is larger, it means that the channel correlation between user i and user j is higher, and thus the interference between the two users is more serious, and vice versa. Therefore, when the absolute value of $coor_{ij}(HV_{i},HV_{j})$ is smaller, the spatial correlation between the two matrices $H_{i}$ and $H_{j}$ is lower.

The relationship between coor and correlation is shown in Table 1 [15]:

The greedy algorithm calculates the noise enhancement factor $l_{k}$ and selects the largest user that can guarantee the maximum ${\sum_{k=1}^{K}\left|l_{k}\right|}^{2}$ of the current system. It can be found that ${\sum_{k=1}^{K}\left|l_{k}\right|}^{2}$ is determined by the channel gain of each user in the set S. Therefore, the user’s noise enhancement factor $l_{k}$ is also a major factor affecting the system capacity. In this paper we define the scheduling factor $\rho_{k}=l_{kk}-\sum_{m\subseteq s}l_{km}l_{mk}$, which measures the conditions of the channel based on the noise enhancement factor with $l_{kk}={\left\|P_{k}^{⊥}H_{k}\right\|}_{2}$ and $P_{{}_{k}}^{⊥}=I-H_{k}^{H}{\left(H_{k}H_{k}^{H}\right)}^{-1}H_{k}$.

In summary, $\rho_{k}coor_{ks}$ is used as the criterion of the multi-user scheduling algorithm in this paper, and the scheduled user set is selected as the effective transmission channel of the system to obtain better system user gain. The expression $coor_{ks}$ represents the Pearson coefficient between the user k in the candidate user set and the selected user set $\hat{H}$.

The specific steps of the above scheduling algorithm are shown in Algorithm 1:
**Algorithm 1 Multi-User Scheduling Based on BD Precoding for UM-MIMO System****Input:** channel matrix H, number of users K, number of users’ antennas $N_{r}$, number of users by the base station R, number of current iterations i=1.**Output:** scheduling user set $\hat{H}$, the number of final scheduling users of the base station M.
Step1: Establish candidate user set S={1, 2, …, K} and selected user set $\hat{H}=\emptyset$;Step2: Traverse the candidate user set **S**, select $s_{1}=\arg\underset{k\in S}{\max\left(C_{k}\right)}$. Update the candidate user set $S=S-\{s_{1}\}$ and the selected user set $\hat{H}=\hat{H}+\{s_{1}\}$, the number of selected users is M=1 with $C_{k}=B\log_{2}\det\left(I_{N_{r}}+\frac{P}{R\sigma^{2}}H_{k}H_{k}^{H}\right)$; Step3: Calculate the channel capacity of the selected user set at this time $C_{\max}=B\log_{2}\det\left(I_{N_{r}}+\frac{P}{R\sigma^{2}}H_{s_{1}}H_{s_{1}}^{H}\right)$;Step4: If the number of iterations is i<R, perform the following steps, otherwise skip to Step10;Step5: Traverse each user in the candidate user set S and calculate the scheduling factor $\rho_{k}$ of each user and the Pearson coefficient $coor_{kL}$ between the user and the selected user set S;Step6: Select users who meet $s_{i}=\arg\underset{k\in S}{\min}\rho_{k}coor_{kL}$ and calculate the system capacity $C_{m}$ currently;Step7: If the current system capacity meets $C_{m}>C_{\max}$, the algorithm continues, otherwise skip to step 10; Step8: Update candidate user set $S=S-\{s_{i}\}$, selected user set $\hat{H}=\hat{H}+\{s_{i}\}$, maxi mum system capacity $C_{\max}=C_{m}$, and user scheduling number M=M+1; Step9: i=i+1, jump to Step4; Step10: The algorithm ends, the scheduling user set $\hat{H}$ is obtained.

## 4. Simulation Results and Analysis

### 4.1. Complexity Analysis

In this section, we compare the complexity of the subspace correlation algorithm [16], geometric angle algorithm [17], conditional number algorithm [18] and the proposed algorithm. The computational complexity is defined as the number of complex operations; complex addition, complex multiplication, and complex division are considered to be complex operations. Therefore, the calculation complexity of various user scheduling criteria is represented by Table 2.

It can be seen from Table 2 that the user scheduling criterion based on matrix vectorization and scheduling factor proposed in this paper is greater than the user scheduling criterion based on subspace correlation. This is mainly because the algorithm proposed in this paper is traversed, so it consumes a certain complexity to improve the performance of the system. Subspace correlation-based algorithms, geometric angle-based algorithms, and condition-based algorithms have the same computational complexity when satisfying $N_{t}\leq N_{r}^{2}$.

### 4.2. Fairness Analysis

In this section, we compare the fairness of the algorithms, as shown in Table 3. Assuming that the dimension of the transmission signal corresponding to each user is equal to the number of receiving antennas, we use the fairness factor to measure the fairness of the algorithm, and define fairness factor as [19]:

(19) $$F\left(i\right)=\frac{{\left(\sum_{i=1}^{k}x_{i}\right)}^{2}}{\sum_{i=1}^{k}x_{i}^{2}}$$

where $x_{i}$ represents the average transmission rate of the user i; F(i) ranges from 0 to 1, and the greater the value, the higher the fairness; F(i)=1 means that users have received the same resources, and the fairness is highest at this time.

As can be seen from Table 3, the fairness of the algorithm proposed in this paper is the highest. This is because our method uses more effective information (channel correlation and channel quality) to implement scheduling, so it performs better in fairness.

### 4.3. System Performance Comparison

In order to verify the feasibility and effectiveness of the algorithm proposed in this paper, and to study the impact of vehicles on the algorithm performance in different mobile environments, this paper uses MATLAB simulation software to build a multi-user ultra-massive MIMO system to simulate the proposed algorithm and the comparison algorithm, and finally to compare the simulation results. In the simulation, it is assumed that the receiving end and the transmitting end can obtain perfect channel state information. There are M users, the number of each user RF chains is $N_{r}^{RF}=1$, and the number of transmitter RF chains is $N_{t}^{RF}=N_{r}^{RF}\times M$. Other main simulation parameters are summarized in Table 4.

This paper mainly compares and simulates the bit error rate (BER) performance and spectrum efficiency of the proposed algorithm and the algorithm based on subspace correlation [16], the geometric angle algorithm [17], and the condition number algorithm [18]. The vehicle speed is 80 km/h, and the vehicle density is determined by the speed of the vehicle.

**Figure 3:**
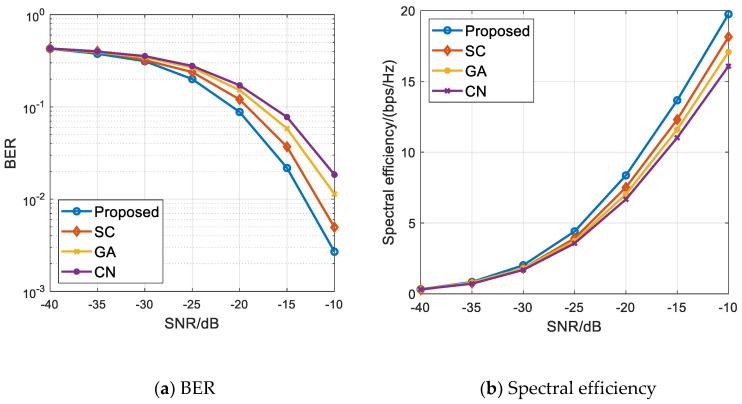
Multi-user scheduling simulation results when M=2 and $N_{t}=256$. (**a**) BER of all the algorithms; (**b**) Spectral efficiency performance of all the algorithms.

Figure 3a,b, respectively, show the BER and spectral efficiency comparison diagrams of the proposed algorithm and the comparison algorithm when 256 transmitting antennas are configured at the base station and two vehicle users are scheduled. We can see from Figure 3 that the BER and channel capacity of the four algorithms are relatively close at low signal-to-noise ratio. With the increase in signal-to-noise ratio, the difference of system BER and total system capacity realized by different scheduling algorithms gradually increases. In general, the proposed algorithm is better than the comparison algorithm in terms of BER and spectral efficiency. This is mainly because the conditions of the channel itself have a great impact on the information transmission effect. There will be a better transmission effect when the conditions of the channel itself are good. The comparison algorithm only considers the correlation of the channel matrix and ignores the influence of the conditions of the channel itself. The algorithm in this paper not only considers the channel correlation, but also considers the conditions of the channel itself. Adding a scheduling factor to measure the conditions of the channel can effectively eliminate the system interference and improve the system performance.

**Figure 4:**
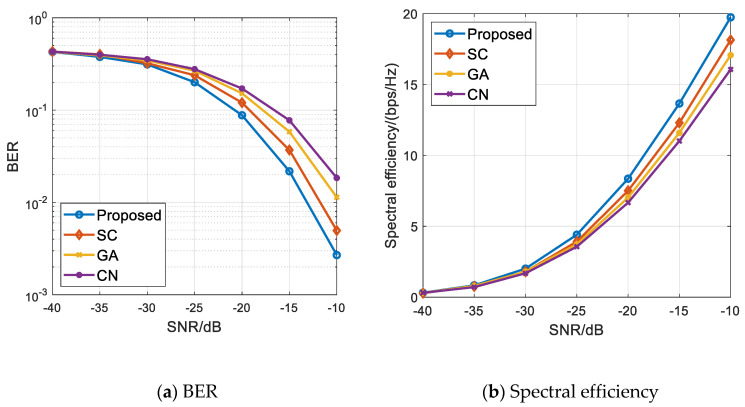
Multi-user scheduling simulation results when M=4 and $N_{t}=256$. (**a**) BER of all the algorithms; (**b**) Spectral efficiency performance of all the algorithms.

Figure 4a,b, respectively, show the BER and spectral efficiency performance comparison diagrams of the above-noted algorithms when 256 transmitting antennas are configured at the base station and four vehicle users are scheduled. We can see from Figure 4 that the capacity difference and BER difference between the proposed algorithm and the other three comparison algorithms are basically the same under the condition of low signal-to-noise ratio in which the number of vehicle user scheduling is four. With the increase in signal-to-noise ratio, the capacity obtained by the proposed algorithm is higher than that of the comparison algorithm, the BER decreases the fastest, and the overall performance of the proposed algorithm is still better than that of the comparison algorithm. This shows that the proposed algorithm can still maintain the optimal performance when the number of scheduling users increases. In addition, a comparative analysis of Figure 4b and Figure 3b shows that the spectrum efficiency achieved by several algorithms in the scenario where the number of vehicle users is four is greater than that of the same algorithm in the scenario where the number of vehicle users is two. This is because with the increase in the number of scheduling users, the system capacity increases, and the transmitter can provide higher diversity gain, so the spectral efficiency performance increases.

**Figure 5:**
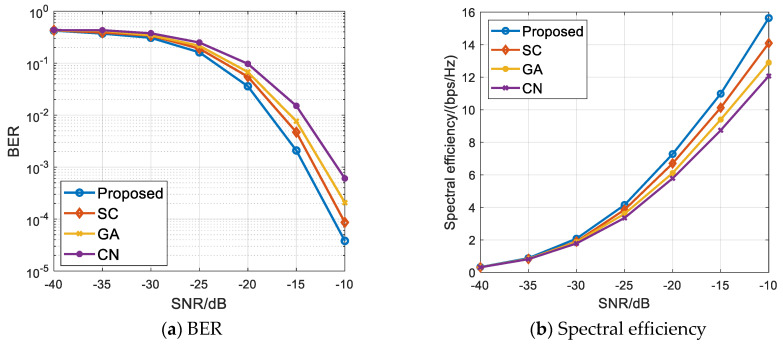
Multi-user scheduling simulation results when M=2 and $N_{t}=128$. (**a**) BER of all the algorithms; (**b**) Spectral efficiency performance of all the algorithms.

Figure 5 shows the comparison of BER and spectrum efficiency performance of the above algorithms when the number of scheduled users is two when 128 antennas are configured at the base station. Compared with Figure 3, the frequency efficiency of the system is also improved when the number of transmitting antennas is increased with the number of scheduling users fixed and the system resources limited. This is because increasing the number of antennas at the transmitting end can improve the antenna array gain and effectively improve the signal-to-interference plus noise ratio (SINR) at the receiving end of the system, to improve the spectrum efficiency of the system. However, with the increase in the number of transmitting antennas, the BER performance of the system decreases. The main reason is that when the number of transmitting antennas increases, the interference between channels also increases, which reduces the effective transmission signal of users. It can also be seen from Figure 5 that the performance of the algorithm proposed in this paper is the best regardless of BER or spectral efficiency. This is because the proposed algorithm not only considers the correlation between channels, but also considers the conditions of the channel itself. This can effectively eliminate the interference between selected users, which also proves that the proposed algorithm is useful in the ultra-massive MIMO system in the 6G V2X scenario.

**Figure 6:**
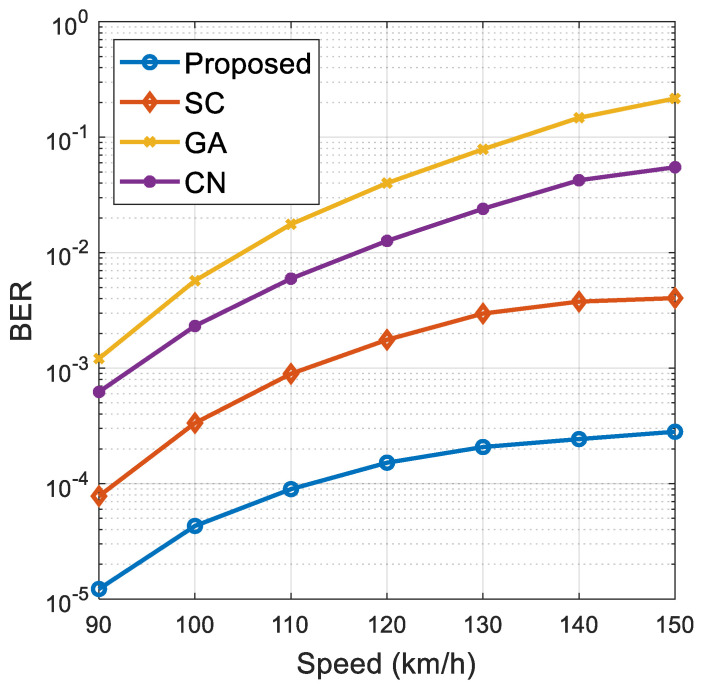
The impact of different vehicle speeds on BER.

**Figure 7:**
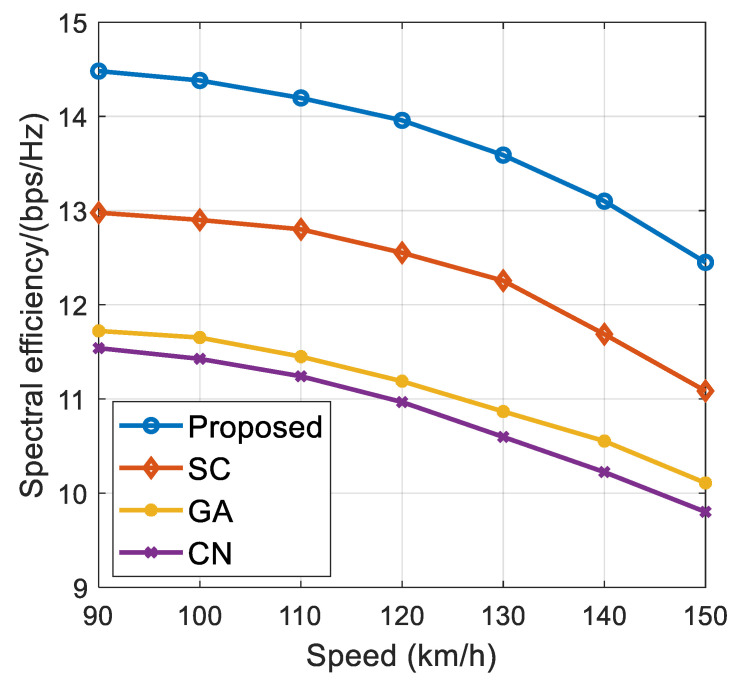
The influence of different vehicle speeds on spectrum efficiency.

Figure 6 and Figure 7, respectively, show the impact of vehicle speed on the proposed algorithm and the performance of the comparison algorithm (including BER and spectral efficiency) under different vehicle speed environments. The vehicle speed is set from 90 km/h to 150 km/h, where SNR = −10 dB, M=2, $N_{t}=128$. It can be seen from Figure 6 and Figure 7 that the BER of the proposed algorithm and the comparison algorithm gradually increases, and the spectral efficiency performance of the system gradually decreases with the continuous increase in vehicle speed. This is because the Doppler effect becomes more serious with an increase in vehicle speed, resulting in the decline of system performance. At the same time, the simulation results show that the proposed algorithm has better robustness than the comparison algorithm. This is because the proposed algorithm makes full use of the time dimension information of the time-varying channel. Therefore, compared with the comparison algorithm, the performance of the proposed algorithm does not decline significantly and has high stability when vehicle speed is increasing.

## 5. Conclusions

This paper proposes a 6G V2X multi-user scheduling algorithm based on BD precoding for ultra-massive MIMO systems. Aiming at the high-speed movement and millimeter wave characteristics of the vehicle in the 6G V2X scheme, a time-varying geometric channel model is established. On this basis, the BD precoding is sensitive to channel correlation, and the Pearson coefficient after matrix vectorization is used to measure the channel correlation between users, comprehensively consider the two factors of channel correlation and channel conditions, and ensure that the channel correlation is minimized while ensuring the selection of high-quality channels to achieve multi-user scheduling. BD precoding is then used to eliminate data stream interference. The system simulation results show that the proposed algorithm can effectively reduce the bit error rate and improve the system spectrum efficiency and is suitable for 6G V2X communication. In future work, we will put the proposed algorithm into actual conditions of automatic driving vehicle communication for verification to observe the stability of the algorithm and better evaluate the support ability of the proposed algorithm in 6G networks to the real automatic driving conditions.

## Figures and Tables

**Table 1:** Correspondence between coor and correlation.

coor	Correlation
0.8<coor≤1.0	Very strong correlation
0.6<coor≤0.8	Strong correlation
0.4<coor≤0.6	Moderately related
0.2<coor≤0.4	Weak correlation
0.0<coor≤0.2	Very weak or no correlation

**Table 2:** Complexity comparison.

Algorithm	Complexity
SC	$O\left(N_{t}N_{r}^{2}\right)$
GA	$O\left(N_{r}^{3}\right)$
CN	$O\left(N_{r}^{3}\right)$
Proposed	$O\left(C_{k}^{M}C_{N_{t}}^{M}N_{r}^{M}\right)$

**Table 3:** Comparison of equity factors (SNR = 20 dB, k = 10).

Scheduling Algorithm	Proposed	SC	GA	CN
Fairness factor	0. 92392	0.87376	0.74418	0.63034

**Table 4:** Main parameter settings of the system simulation.

Simulation Parameters	Settings
Carrier frequency	28 GHz
$\mathrm{Transmit}\;\mathrm{antenna}N_{t}$	256/512
Number of single-user receiving antennas $N_{r}$	8
Number of single user data streams $N_{s}$	2
User number M	2/4
Channel model	Time-varying geometric channel
$\mathrm{Number}\;\mathrm{of}\;\mathrm{clusters}N_{c}$	3
$\mathrm{Number}\;\mathrm{of}\;\mathrm{paths}\;\mathrm{in}\;\mathrm{each}\;\mathrm{cluster}N_{ray}$	7
Azimuth mean distribution	[0,2π]
Mean elevation angle distribution	[−2/π,2/π]
Antenna array structure	ULA
Antenna spacing d	$0.5^{\lambda }$
Vehicle antenna height	1.5 m
Vehicle density	0.0025/0.005/0.0083 vehicle/m
Angle expansion	7.5°
Vehicle moving direction	Move in a straight line along the road
Channel estimation	Ideal

## Data Availability

The data used to support the findings of this study are available from the corresponding author upon request.
